# Fisetin Deters Cell Proliferation, Induces Apoptosis, Alleviates Oxidative Stress and Inflammation in Human Cancer Cells, HeLa

**DOI:** 10.3390/ijms23031707

**Published:** 2022-02-01

**Authors:** Nazia Afroze, Sreepoorna Pramodh, Jasmin Shafarin, Khuloud Bajbouj, Mawieh Hamad, Madhumitha Kedhari Sundaram, Shafiul Haque, Arif Hussain

**Affiliations:** 1 School of Life Sciences, Manipal Academy of Higher Education-Dubai Campus, Dubai P.O. Box 345050, United Arab Emirates; afroze.nazia@gmail.com (N.A.); madhumithakedhari@gmail.com (M.K.S.); 2 Department of Life and Environmental Sciences, College of Natural and Health Science, Zayed University, Dubai P.O. Box 19282, United Arab Emirates; Sreepoorna.Unni@zu.ac.ae; 3 College of Medicine, University of Sharjah, Sharjah P.O. Box 27272, United Arab Emirates; jsalam@sharjah.ac.ae (J.S.); kbajbouj@sharjah.ac.ae (K.B.); mabdelhaq@sharjah.ac.ae (M.H.); 4 Research and Scientific Studies Unit, College of Nursing and Allied Health Sciences, Jazan University, Jazan P.O. Box 114, Saudi Arabia; shafiul.haque@hotmail.com; 5 Faculty of Medicine, Görükle Campus, Bursa Uludağ University, Nilüfer 16059, Turkey

**Keywords:** fisetin, cytotoxicity, glutathione, AKT/mTOR, MAPK, JAK-STAT/NF-kB, phosphorylation

## Abstract

Background: Fisetin, a flavonol profusely found in vegetables and fruits, exhibited a myriad of properties in preclinical studies to impede cancer growth. Purpose: This study was proposed to delineate molecular mechanisms through analysing the modulated expression of various molecular targets in HeLa cells involved in proliferation, apoptosis and inflammation. Methods: MTT assay, flow cytometry, nuclear morphology, DNA fragmentation and Annexin–Pi were performed to evaluate the anti-cancer potential of fisetin. Furthermore, qPCR and proteome profiler were performed to analyse the expression of variety of gene related to cell death, cell proliferation, oxidative stress and inflammation and cancer pathways. Results: Fisetin demonstrated apoptotic inducing ability in HeLa cells, which was quite evident through nuclear morphology, DNA ladder pattern, decreased TMRE fluorescent intensity, cell cycle arrest at G_2_/M and increased early and late apoptosis. Furthermore, fisetin treatment modulated pro-apoptotic genes such as APAF1, Bad, Bax, Bid and BIK at both transcript and protein levels and anti-apoptotic gene Bcl-2, BIRC8, MCL-1, XIAP/BIRC4, Livin/BIRC7, clap-2/BIRC3, etc. at protein levels to mitigate cell proliferation and induce apoptosis. Interestingly, the aforementioned alterations consequently led to an elevated level of Caspase-3, Caspase-8 and Caspase-9, which was found to be consistent with the transcript and protein level expression. Moreover, fisetin downregulated the expression of AKT and MAPK pathways to avert proliferation and enhance apoptosis of cancer cells. Fisetin treatment also improves oxidative stress and alleviates inflammation by regulating JAK-STAT/NF-kB pathways. Conclusion: Together, these studies established that fisetin deters human cervical cancer cell proliferation, enhances apoptosis and ameliorates inflammation through regulating various signalling pathways that may be used as a therapeutic regime for better cancer management.

## 1. Introduction

Cancer is a pleiotropic disease caused by unorchestrated and undesirable cell reproduction. It is the second leading cause of mortality worldwide, followed by cardiac ailments. The undesirable side effects of various conventional treatments, including chemotherapy and chemopreventive agents such as Tamoxifen aspirin, sulindac, Raloxifene, etc. along with lack of specificity, confines the generous use of these treatments; therefore, researchers in the last two decades have shifted their focus towards phytochemicals (plant-derived chemopreventive agents) that exhibit a differential response against transformed cells with a safer profile [1,2]. Comprehensive epidemiological data demonstrate a strong correlation between a diet rich in fruits and vegetables and the reduced threat of carcinogenesis, as they are enriched with polyphenols (which contains multiple phenolic groups) [3,4]. 

Dietary polyphenols possess a repertoire of biological properties with remarkable cancer chemopreventive and therapeutic potential against different types of cancers by targeting various hallmarks of cancer and modulating their activities [5,6,7]. Several studies have established that flavonoids such as EGCG, resveratrol, curcumin, genistein, quercetin, luteolin, sulforaphane, etc., deter cancer growth, reduce inflammation, improve anti-oxidation and induce apoptosis by modulating various apoptotic proteins and signalling pathways such as MAPK, NF-kB, PI3K/Akt, Wnt, etc. [8,9,10,11]. Flavonoid exposure to different cancer cell lines has differentially demonstrated apoptosis, which is facilitated either by extrinsic (death receptor pathway) or intrinsic pathway (mitochondrial pathway), or both. The death receptor pathway is mediated by TNF-receptors including TRAIL-R2, TRAIL-R1, TNFRI and Fas, which are alternatively known as DR5, DR4, DR1 and DR2, respectively. The anti-survival molecules such as Bad, Bak, Bax, and pro-survivals Bcl-XL and Bcl-2 are involved in the intrinsic pathway [12,13,14].

**Figure 1:**
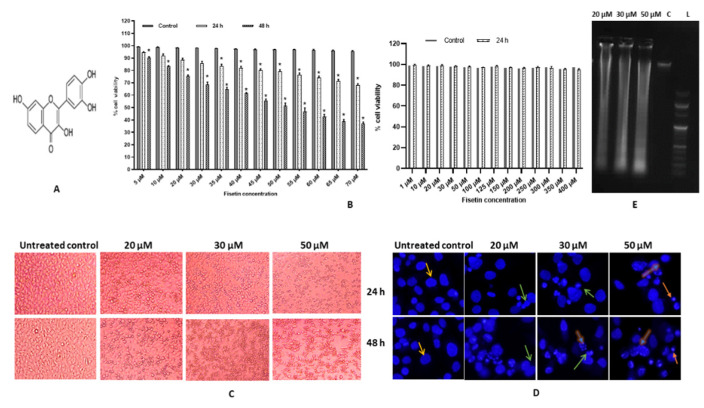
Cytotoxic effects of fisetin on HeLa cells. (**A**) Chemical structure of fisetin. (**B**) Graph represents dose and time-dependent decrease in cell viability of HeLa cells after treatment with fisetin [1–70 µM] for 24 h and 48 h, respectively, whereas fisetin did not demonstrate any significant difference in the cell viability of AC-16 (normal cell line). All the assay-treated cells were compared with DMSO controls. The IC50 of fisetin was found to be 50 µM at 48 h. The data are expressed as the mean ± standard deviation of three independent experiments. Statistically significant differences are marked by asterisks: two-way ANOVA * represents *p* < 0.05; (**C**) Microscopic examination of treated cells: Fisetin treated HeLa cells at various concentrations [20, 30 and 50 µM] and time points [24 h and 48 h] show the characteristic feature of rounding off of the cells, signifying apoptosis at 10X magnification. (**D**) Nuclear morphology of fisetin treated HeLa cells [20, 30 and 50 µM] shows dose-dependent increase in apoptotic index. Orange = prominent intact nuclei, green = membrane blebbing, yellow = nuclear fragmentation, green = apoptotic bodies. (**E**) HeLa cells treated with different concentrations [20, 30, 50 μM for 48 h] of fisetin were found to produce a DNA laddering pattern consistent with apoptosis. C = DMSO Control, L = DNA ladder.

Fisetin (3,7,3,4-tetrahydroxyflavone), a flavonol (sub-class of polyphenol), is found profusely in vegetables and fruits such as tea, onion, cucumber, apple, strawberries, etc., and exhibits a broad range of biological properties such as anti-inflammatory, antioxidant, apoptosis-inducing, anti-migratory, neuroprotective, anti-cancer, etc. (Figure 1A) [3,15].

Fisetin demonstrates anti-metastatic properties by suppressing phosphoinositide 3-kinase/Akt [PI3K/Akt], NF-kB, and JNK signalling pathways in prostate cancer cells and human cervical cancer HeLa cells [4,16]. It acts as an inhibitor of PI3K/Akt, which is found to be overexpressed in different types of cancers to impede cancer growth of prostate, pancreas and lung cancer [10]. Fisetin enhances apoptosis in different cell lines by changing the quotient of Bax/Bcl-2 and upregulation of Caspase 3, 8 and 9 activities. Fisetin also inhibits cell proliferation by arresting cell cycle at G2/M phase in A 431 cells (human epidermoid carcinoma) [17,18,19,20,21,22]. Moreover, fisetin also possesses a potent protective property as it relieves the oxidative stress and inflammation by upregulating different enzymes such as glutathione peroxidase, catalase, superoxide dismutase, etc., and decreases the expression of pro-inflammatory cytokines or increases anti-inflammatory cytokines [23,24,25].

This study is aimed to investigate anti-proliferative, anti-apoptotic, antioxidant and anti-inflammatory properties with an insight into the molecular mechanism involving signalling pathways that are being modulated by fisetin.

## 2. Results

### 2.1. Fisetin Induces Morphological Changes and Inhibits Proliferation of HeLa Cells

The MTT test was employed to investigate fisetin’s cytotoxic effects on HeLa cell proliferative capacity. Fisetin-treated HeLa cells from 1–70 μM for 24 and 48 h displayed an inhibitory effect on growth of HeLa cell both with increasing time and concentration-dependent manner which ranged from 4–32% in 24 h and 18–67% in 48 h (*p* < 0.001). The IC_50_ value was 50 μM at 48 h. Fisetin did not show any significant change in cell viability of AC-16 (cardiomyocyte; normal cell line), thus illustrating differential cytotoxic, hence a safer profile (Figure 1B). All the experiments were repeated at least thrice. The morphology of the treated cells was different in comparison to the control, and they were rounded off and detached from the surface. The percentage of dead cells at 20, 30 and 50 µM increased both in time and dose-dependent manner (Figure 1C).

### 2.2. Fisetin Changes Nuclear Morphology of HeLa Cells

To evaluate the changes in nuclear morphology, treated cells with 20, 30 and 50 µM of fisetin were stained with DAPI. DAPI staining demonstrated that fisetin treated cells revealed nuclear condensation, fragmentation and apoptotic body formation, which was augmented with both concentration and time-point modes (Figure 1D).

### 2.3. Fisetin Leads DNA Fragmentation

To evaluate the mechanism of fisetin-induced cytotoxicity, DNA fragmentation assay was performed. DNA analysis of the fisetin-treated HeLa cells (20 μM and 50 μM) for 48 h demonstrated that fisetin efficiently reduces DNA integrity and consequently induces DNA ladder formation in treated cells in a dose-dependent manner as evident via agarose gel electrophoresis. In contrast, the DNA of the control cells remained intact (Figure 1E). 

### 2.4. Fisetin Encourages G2/M Arrest and Modulates Cell Cycle Regulatory Genes

**Figure 2:**
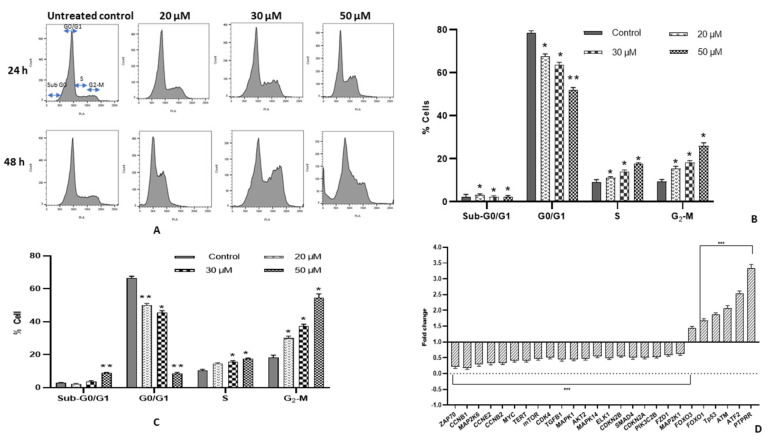
(**A**) Flow cytometry analysis: Analysis of DNA content of treated HeLa cells with 20, 30 and 50 µM of fisetin for 24 and 48 h was compared with DMSO control cells, after PI staining. It demonstrated G2/M arrest cell cycle arrest with increase in sub-G0 apoptotic population. (**B**,**C**) Graph represents % distribution of cells across the different phases of cell cycle in 24 and 48 h, respectively. (**D**) RQ plot of HeLa cells followed by treatment with fisetin for 48 h resulted in downregulation of various cell cycle regulators, genes involved in PI3K/AKT, MAPK and WNT signalling, while upregulation in TSGs expression compared with the control. The data are expressed as the mean ± standard deviation of three independent experiments. Statistically significant differences are marked by asterisks: two-way ANOVA * represents *p* < 0.05; ** represents *p* < 0.01.

Fisetin affects the proliferation of HeLa cells via cell cycle arrest and causes apoptosis. Treated cells (20, 30 and 50 μM) for 24 and 48 h and the untreated control were subjected to flow cytometry. Treated cells with 20, 30 and 50 μM fisetin resulted in a significant accumulation of cells in G2/M phase (Figure 2A). It has increased from 10.1% to 16.2%, 18.9%, and 25.1%, respectively in 24 h while in 48 h the proportion of G2/M arrested cells increased to 30.9%, 36.2%, 56.2% at 20 μM, 30 μM and 50 μM, respectively. Simultaneously, a significant proportion of cells was built up in Go/G1 phase at 50 μM of fisetin at 48 h (Figure 2B,C).

Fisetin-mediated G2/M cell cycle arrest was additionally substantiated by evaluating modulation in genes manifestation involved in cell cycle regulation, after treatment at 50 μM only. Fisetin illustrated downregulation of *CCNB1, CCNB2, CCNE2, CDK2 and, CDK4* corresponding to G2/M phase arrest and TERT enzyme, which deters cell proliferation. In contrast, *PTPRR, FOXO1, FOXO3* expression was elevated. PTPRR acts as an ERK/MAPK pathway inhibitor, while the latter two play a significant role in the extrinsic apoptotic pathway by upregulating FasL and TRAIL (pro-apoptotic factors) (Table 1) (Figure 2D). 

### 2.5. Fisetin Shows Early Apoptosis on HeLa Cells

**Figure 3:**
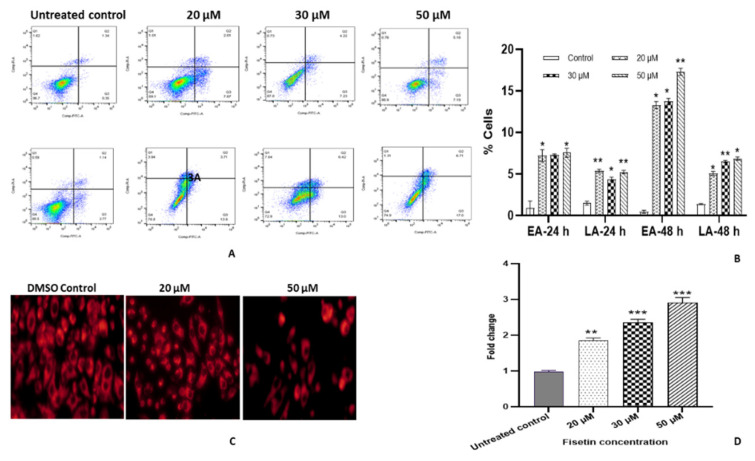
Fisetin induces apoptosis in HeLa cells (**A**) Fisetin-treated HeLa cells with 20 µM, 30 µM and 50 µM for 24 and 48 h in comparison with the DMSO control followed by double staining. Representative picture of dot plots showing different stages of apoptosis. Left lower quadrant (FITC^−^/PI^−^) = viable cells, right lower quadrant (FITC^+^/PI^−^) = early apoptotic cells, right upper quadrant (FITC^+^/PI^+^) = late apoptotic cells. (**B**) Graph illustrating the percentage distribution of different stages of apoptotic cells in their respective quadrant by flow cytometry. Early and late apoptotic cell proportions was found to be increased both in time and concentration-dependent manner compared with the control. (**C**) TMRE staining of treated cells showing reduction in fluorescent intensity signifying reduction in mitochondrial membrane potential. Images were captured by fluorescent microscope. (**D**) Graph representing TMRE fluorescence of treated HeLa cells with fisetin 20, 30 and 50 µM for 48 h, which exhibited reduction in mitochondrial membrane potential from 81% to 64% and 54%, respectively, in comparison with the untreated control. Data are presented as the mean ± standard deviation of three independent experiments. Two-way ANOVA * = *p* < 0.05; ** = *p* < 0.01, *** *p* < 0.001.

Annexin V/PI double staining on treated HeLa cells at 20, 30 and 50 μM for 48 h displayed an increase in the early and late apoptotic cells, whereas the percentage of live cells showed a decreasing trend. Fisetin treatment resulted in the augmentation of both early and late apoptotic cell populations. Early apoptotic cell population was found to be increased from 0.35% to 13%, 13.6% and 17%, likewise, PI-stained post apoptotic cells increased in proportion from 1.34% to 3.71%, 6.42% and 6.71% at 20, 30 and 50 μM, respectively. Therefore, Annexin–PI staining established the apoptosis-inducing property of fisetin in HeLa cells (Figure 3A,B).

### 2.6. Fisetin Decreases TMRE Fluorescent Intensity

Altered mitochondrial potential is one of the triggers for apoptotic induction. The mitochondrial membrane potential of the untreated control was compared with the fisetin-treated cells to investigate whether fisetin induces mitochondrial dysfunction to release cyt-c. After staining the treated and untreated control cells with TMRE for half an hour, washed cells were examined under an inverted fluorescent microscope at ×40. A bright red fluorescent aggregate was observed in the mitochondrial matrix of untreated control, whereas the fluorescent intensity was found to be decreasing with the increasing concentration of fisetin (20, 30 and 50 μM) treated cells from 74% to 70% and 54% (Figure 3C,D).

### 2.7. Fisetin Activates Extrinsic and Intrinsic Pathways

**Figure 4:**
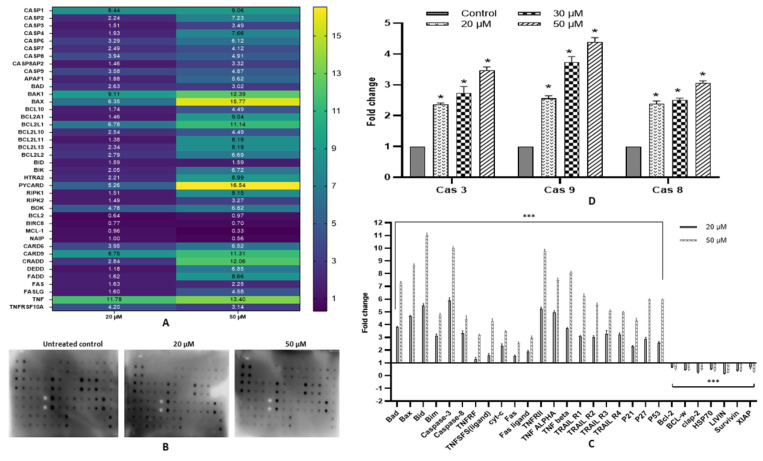
Expression analysis. (**A**). Heat map showing the expression of various genes involved in apoptosis. RQ plot of caspases, extrinsic receptors and ligands, pro-apoptotic gene and anti-apoptotic after fisetin treatment at 20 μM and 50 μM for 48 h. (**B**) Images of nitrocellulose proteome profiler showing differential expression of the regulatory pathway and apoptotic proteins in the control and fisetin-treated sample (20 and 50 μM of fisetin for 48 h). (**C**) Graphical representation of protein expression as fold change compared with the control sample. Fisetin treatment increased pro-apoptotic while decreasing the expression of anti-apoptotic proteins [* *p* ≤ 0.05, *** *p* < 0.001]. (**D**) Evaluation of caspase 3, caspase 8 and caspase 9 activity of fisetin-treated HeLa cells at 20, 30 and 50 μM for 48 h. Graph represents an increase in the fold change in caspase 3, 8 and 9 activity compared with the control.

The apoptosis-inducing property of fisetin was substantiated by detecting an alteration in the expression of various genes pertaining to death receptor and the intrinsic (mitochondrial pathway) apoptotic pathway. Fisetin treated cells with 20 and 50 µM for 48 h displayed upregulation of various pro-apoptotic genes, whereas anti-apoptotic genes were downregulated. The expression of multiple receptors and ligands involved in extrinsic pathways such as FAS, *FASL,* CARD6, CARD9, CRADD, DEDD, *FADD, TNF, TNFRSFS10A and* *TNFRSFS10B* increased, which, as well as the upregulation of *Caspases8Ap2* and *Caspase8,* are indicative of the extrinsic pathway of apoptosis. Genes of *BCL2* family such as *BCL10, BCL2A1, BCL2L1, BCL2L13, BCL2L2, BAD, BAK1* and *BAX, BID, BOK*, HTRA2, PYCARD, RIPK1, RIPK2 and *BID* were also upregulated significantly, and the apoptotic inhibitory proteins such as *Bcl-2, MCL1, BIRC5 and NAIP* were discovered to be under expressed. Caspases such as *caspase 1,* *caspase 2, caspase 3, caspase 4, caspase 6, caspase 7* *and* *caspase 9* also exhibited upregulation along with *APAF1* at the transcript level, therefore signifying an intrinsic pathway (Table 1) (Figure 4A).

### 2.8. Fisetin Modulates Expression of Various Pro- and Anti-Apoptotic Proteins

Fisetin treated HeLa cells showed dose-dependent modulation of different proteins involved in apoptosis, a trend that concords with the transcript expression. The expression of Bad, Bax, Bid, Bim, P21, p53, p27, TNFRF, TNFSFS (ligand), cyt-c, Fas, Fas ligand, HSP27, IGFBP-1 to IGFBP-4, TNFRII, TNF ALPHA, TNF BETA, TRAIL R1 to TRAIL R4, caspase-3 and caspase-8 were upregulated. In contrast, the pro-survival proteins such as Bcl-2, BCL-w, clap-2, HSP70, LIVIN, Survivin and XIAP expression were found to be significantly decreased (Figure 4B,C).

### 2.9. Fisetin Elevates Caspase-3, Caspase-8 and Caspase-9 Activity

Fisetin-treated cells were analysed for caspase-3, -8 and -9 expression by a fluorometric assay. Fisetin treatment at 20, 30 and 50 μM for 48 h showed comparative dose-dependent elevation in caspase- 3 and Caspase-9 activity from 2.3 to up to 4.4-fold and from 2.5 to little more than 4.5-fold, respectively, whereas Caspase-8 displayed an upregulation of up to 2.9-fold only (Figure 4D).

### 2.10. Fisetin Ameliorates Oxidation Stress in HeLa Cells by Upregulating GSH Activity

**Figure 5:**
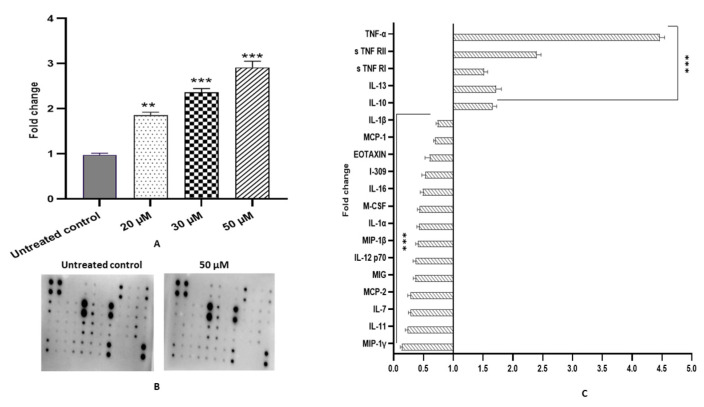
Analysis of inflammatory cytokines (**A**) Nitrocellulose membrane showing the differential expression of inflammatory cytokines (**B**) Graph showing the downregulation of pro-inflammatory and chemokines while showing upregulation in anti-inflammatory cytokines expression in fisetin treated (50 µM) compared with the DMSO control sample. (**C**) Graph showing an increase in total GSH level in fisetin-treated HeLa cells at 20, 30 and 50 μM for 48 h. Data are presented as the mean ± standard deviation of three independent experiments. Two-way ANOVA. ** = *p* < 0.01, *** *p* < 0.001.

Fisetin-treated cells (20, 30, and 50 μM for 48 h) exhibited upregulation in GSH level to 1.8, 2.4, and 3-fold, respectively, compared with the DMSO control (Figure 5A).

### 2.11. Fisetin Alleviates Inflammation in HeLa Cells

Carcinogenesis induced by inflammation correlates with oxidative stress, imbalanced cytokine production and a modulated NF-κB signalling pathway. Fisetin-treated HeLa cells at 50 μM for 48 h demonstrated downregulation in the expression of various inflammatory cytokine proteins such as IL-1α, IL-1β, IL-4, IL-7, IL-11, IL-16, IL-12p70, MIG, MCP-1, MCP-2, MIP-1β, MIP-1γ, MCF, I-309 and EOTAXIN compared with untreated control cells, whereas the expression of IL-10 and IL-13 were reduced (Figure 5B,C). 

### 2.12. Fisetin Changes the Aberrant MAPK and PI3K/AKT/mTOR in HeLa Cells

**Figure 6:**
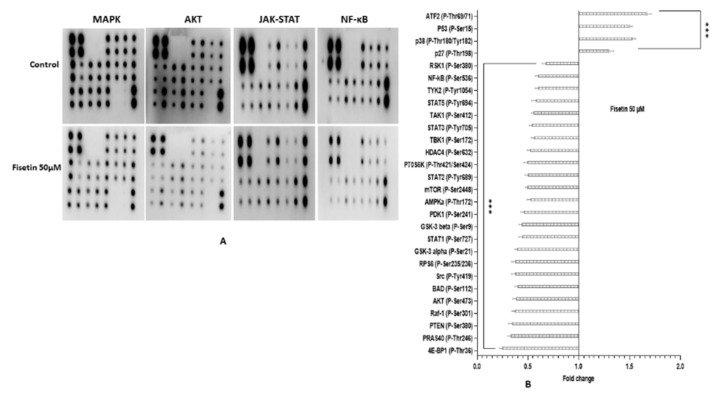
Differential expression of various phosphorylated proteins associated with different signalling pathways. (**A**) Images of proteome profiler membranes showing differential expression of the phosphorylated proteins involved in MAPK, AKT, JAK-STAT, NF-ĸB and TGFβ signalling pathway after 50 μM of fisetin treatment for 48 h in comparison with the DMSO control. (**B**) Graphical presentation of downregulated expression of different proteins in the aforementioned pathways, while the expression of P53 (p-ser241) and P27 (p-Thr198), p38(P-Thr180/Tyr-182) were upregulated. Differential expression is shown as fold change. Data are presented as the mean ± standard deviation of three independent experiments *** *p* < 0.001.

Carcinogenesis is correlated with the deviant stimulation of various genes of MAPK, AKT/mTOR and TGF-β pathways that may subsequently cause enhanced cell proliferation and inhibition of apoptosis. In total, 50 μM of fisetin treated HeLa cells for 48 h demonstrated downregulation in the expression of different genes involved in the MAPK pathway such as MAPK1, MAK14, MAP2K1, MAP2K6 and ELK 1 along with AKT/MTOR pathway genes such as AKT2, MTOR, PIK3C2B and PIK3CB. In contrast, the expression of ATM, ATF2, and VHL was increased, consequently decreasing MYC expression (Figure 2D). Moreover, fisetin modifies various phosphorylated proteins associated with cancer pathways. Fisetin (50 μM) treatment altered the expression of phosphorylated proteins involved in MAPK, AKT, JAK-STAT and NF-kB. MAPK pathway genes at the protein level exhibiting downregulation in expression are RSK1 (P-Ser380) and Raf-1 (P-Ser301), whereas p38 (P-Thr180/Tyr182) and P53 (P-Ser15) were found to be upregulated. The phosphorylated protein of the AKT pathway, such as GSK3a (p-ser21), GSK3b (p-ser9), MTOR (p-ser2448), PRAS 40 (p-Ther246), BAD (p-ser112), PTEN (p-ser380), AKT (p-ser473), AMPKa (P-Thr172), RPS6 (P-Ser235/236) and 4E-BP1 (P-Thr36) showed reduced expression while p27 (P-Thr198) was increased compared with the control. Similarly, fisetin exhibited its potential anti-inflammatory action by downregulating both JAK-STAT and NF-kB pathways. The phosphorylation level of various molecular targets was altered, such as Src (P-Tyr419), STAT1 (P-Ser727), STAT2 (P-Tyr689), STAT3 (P-Tyr705), STAT5 (P-Tyr694), TYK2 (P-Tyr1054), HDAC4 (P-Ser632), NF-kB (P-Ser536), TAK1 (P-Ser412) and TBK1 (P-Ser172). HDAC2 (P-Ser394) phosphorylation was also reduced marginally (Figure 6A,B). 

## 3. Discussion

Fisetin displays multifaceted biological activity against different types of cancer, whose impressive anticancer activity was established by an overwhelming number of studies [19,26,27,28]. Potential anticancer properties exhibited by fisetin include anti-proliferation, tumour growth arrest by the modulation of essential factors that regulate the cell cycle and apoptosis. The apoptosis-inducing property of fisetin in transformed cells is the key feature of a potential chemopreventive agent. The current study analysed and verified that fisetin inhibits proliferation and acts as an apoptotic inducer in HeLa cells in in vitro conditions. This study shows that fisetin reduces the viability of HeLa cells in both concentration and time-dependent modes, the IC_50_ value was determined as 50 µM at 48 h. Consistent with other studies that demonstrated similar results, IC_50_ value was documented in previous studies to be IC_50_ of 52 ± 0.9 and 36 ± 0.5 µM at 24 and 48 h, respectively. In lung adenocarcinoma, A549 cell line was 58 µM in 48 h [14], in breast adenocarcinoma, MDA-MB-231 -78 and -68 µM in 24 and 48 h, respectively [16], in Squamous carcinoma, A431 58 µM and 50 µM in 24 and 48 h [20]. Additionally, fisetin illustrated differential cytotoxic as it did not exhibit cytotoxicity towards AC-16 cells (Figure 1B). Treated cell morphologies were distinct compared with the DMSO control as they were rounded off and floating in the media. The percentage of dead cells at 20, 30 and 50 µM increased both in time and dose-dependent manner (Figure 1C). The safer profile of fisetin and other polyphenols such as quercetin, luteolin in normal cell lines and animal models was reported in other studies as well [13,29,30]. The differential cytotoxic action of fisetin towards tumour/cancer cells marks it as an ideal drug candidate.

Nuclear morphological alteration followed by fisetin treatment (20, 30 and 50 µM) of HeLa cells through DAPI staining showed characteristics of apoptotic nuclear morphology that proves the apoptosis-inducing property of fisetin, which showed an increasing trend both in the dose and time-dependent manner. The treated cells displayed membrane blebbing, chromatin condensation and apoptotic body formation, whereas no significant change was observed in the nucleus of untreated control cells. Similar results were reported in glioma, HeLa cells, etc. (Figure 1D) [13,31,32]. Furthermore, fisetin treatment at 20, 30 and 50 µM for 48 h led to inter-nucleosomal degradation of DNA, which upon gel electrophoresis demonstrated a ladder pattern formation. The degradation was found to be increasing with increasing concentration, whereas the DNA of the untreated control did not undergo any degradation (Figure 1E). Flavonoids such as fisetin, chrysin and quercetin were shown to trigger apoptosis through DNA fragmentation in different cell lines [3,32,33]. The DNA content of treated cells at 0, 20, 30 and 50 µM was ascertained by flow cytometry to illustrate the anti-proliferative property of fisetin, as cell cycle checkpoints are the regulatory points for cell growth. Fisetin treatment in HeLa cells demonstrated an accumulation of cells that was dose dependent in G2/M phase from 10.1% to 16.2%, 18.9% and 25.1%, respectively, in 24 h. In comparison, at 48 h, the proportion of G2/M-arrested cells increased to 30.9%, 36.2% and 56.2% at 20, 30 and 50 μM, respectively, and a significant proportion of cells was also built up in Go/G1 phase at 50 μM of fisetin at 48 h (Figure 2A–C). Several reports confirmed that flavonoids exhibit their anti-proliferative property by arresting cell cycle at G2/M [33,34]. This study aligns with the previous studies which confirmed fisetin causes G2/M cell cycle arrest in A431 cells and hepatocellular carcinoma HepG2 cell line while Go/G1 arrest in bladder cancer cell lines T24 and prostate cancer cell line PC3, LNCa [16,35]. In addition, G_2_/M arrest was further established at molecular level by analysing the transcript-level expression of different cell cycle regulatory genes. Molecular-level study results demonstrate consistency with the result found in flow cytometry as fisetin treatment downregulated the expression of *CCNB1, CCNB2, CCNE2, CDK2, CDK4 and TERT* that corresponds to G2/M phase arrest [18,19]*. hTERT* is responsible for maintaining the integrity and stability of linear chromosome. Therefore, its inhibition by polyphenols aids in halting cell cycle progress and preventing cell proliferation. In addition, fisetin treatment led to the elevated expression of *PTPRR, FOXO 1* and *FOXO 3* [Table 1]. FOXO1/3 are tumour suppressor genes [TSG] that upregulate transcription of various genes involved in anti-survival pathways such as FasL and TRAIL, and DNA repair and cell cycle regulation. Expression of these TSGs was reported to be decreased in several cancer cell lines. PTPRR acts as an inhibitor of the ERK/MAPK pathway. therefore helping to deter cell propagation [13,30,36,37,38] (Figure 2D).

Assumption of fisetin-mediated apoptosis was further evaluated by Annexin V/Propidium Iodide double staining. After 48 h of fisetin treatment, the early apoptotic cell population was found to be increased from 2.77% to 13%, 13.6% and 17%, likewise, the PI-stained post apoptotic cell population increased in a dose-dependent manner from 1.14% to 3.71%, 6.42% and 6.71% at 20, 30 and 50 μM, respectively [14,19] (Figure 3A,B). Furthermore, apoptosis was confirmed through disruption of mitochondrial potential using TMRE dye. The fluorescent intensity was evidenced to be decreased from 75% to 70% and 54% with the increasing concentration of fisetin-treatment i.e. 20, 30 and 50 μM respectively, compared to untreated control. Earlier studies confirmed that fisetin leads to depolarization of the mitochondrial membrane to induce apoptosis in the gastric cancer cell and HeLa cells [19,30,32] (Figure 3C,D).

Anti-survival and pro-survival genes are principal molecules pertaining to the regulation of cell death pathways, and their aberrant manifestation drives the cells towards tumorigenesis; therefore, respective modulation of these genes via chemopreventive agents can be crucial to mitigate cancer growth and provide a safer treatment opportunity [14,19,20,30]. Fisetin-treated cells with 20 and 50 µM for 48 h resulted in upregulation of various pro-apoptotic genes, whereas anti-apoptotic genes were downregulated. The expression of multiple receptors and ligands involved in extrinsic pathways such as *FAS,* *FASL,* *CARD6*, *CARD9, CRADD, DEDD*, *FADD, TNF, TNFRSFS10A* and *TNFRSFS10B* increased, that, as well as the upregulation of *Caspases8Ap2* and *Caspase8,* are indicative of extrinsic pathways of apoptosis. Genes of the *BCL2* family such as *BCL10, BCL2A1, BCL2L1, BCL2L13, BCL2L2, BAD, BAK1* and *BAX, BID, BOK*, *HTRA2, PYCARD, RIPK1, RIPK2,* and *BID* were also upregulated significantly, and the apoptotic inhibitory proteins such as *BCL2, MCL1, BIRC5,* and *NAIP* were discovered to be under regulated. Both initiator and executioner caspases involved in intrinsic pathways such as *caspase 1,* *caspase 2, caspase 3, caspase 4, caspase 6, caspase 7,* and *caspase 9* also exhibited upregulation along with *APAF1* at the transcript level, therefore signifying an intrinsic pathway (Figure 4A). In addition, concordant with the transcript level expression, apoptotic proteins also exhibited an increasing trend in concentration-dependent manner such as the expression of Bad, Bax, Bid, Bim, P21, p53, p27, TNFRF, TNFSFS (ligand), cyt-c, Fas, Fas ligand, HSP27, TNFRII, TNF α, TNF β, TRAIL R1 to TRAIL R4, caspase-3 and caspase-8 were upregulated, whereas the anti-apoptotic proteins such as Bcl-2, BCL-w, clap-2, HSP70, LIVIN, Survivin and XIAP expression were found to be significantly decreased (Table 1). Similar results were previously described by fisetin on different cell lines [21,22,27,39,40] (Figure 4B,C). Modulation in expression of aforementioned molecules at transcript and protein level was also induced by other flavonoids [13,30]. Furthermore, fisetin-mediated transcript and protein level increases in caspase 3, 8 and 9 expressions were validated by biochemical activity of these caspases. In addition, concordant with mRNA and protein level, the biochemical activity of caspase 3, 8 and 9 (at 20, 30 and 50 μM) was increased by fisetin ≤ 4, 3.5 and 5-fold, respectively [19,27] (Figure 4D). Apoptosis is mediated by various caspases, of which Caspase 8 is involved in the extrinsic pathway along with death receptors, while caspase 9 in the intrinsic pathway and expression of both the caspases was increased by fisetin along with Fas, Fas ligand, HSP27, TNFRII, TNF α, TNF β and TRAIL R1 to TRAIL R4. Caspase 3 is the executioner caspase, which is common in both extrinsic and intrinsic pathways, therefore confirming that fisetin mediates apoptosis via both death receptor and mitochondrial-mediated pathways. The result of this study are consistent with available reports that have documented that fisetin induces apoptosis by increasing the activity of caspase 3, 8 and 9 [19,33,41].

The anti-oxidative system protects organisms from the impairment initiated by oxidative stress consequently caused by free radicals. Disparity between the oxidation–antioxidation system has been reported in various cancers, including cervical cancer [42,43]. Various reports established a strong association between oxidative stress and inflammation. Chronic inflammation produces a variety of ROS (free radicals) such as superoxide (O_2_^−^), singlet oxygen (^1^O_2),_ nitric oxide (NO) and hydrogen peroxide (H_2_O_2_), which were established to cause oxidative stress and genetic damage to the cervical epithelium, which leads to their conversion into cancer cells [41,44,45]. Therefore, inflammation and oxidative stress are viewed as two of the major factors that lead to carcinogenesis.

Various endogenous enzymes such as superoxide dismutase (SOD), glutathione peroxidase (GTPx), catalase (CAT) and glutathione transferase [GST] protects the cells from oxidative stress as they can scavenge free radicals, thereby regulating the activity of various enzymes and proteins [44]. Flavonoids such as myricetin, quercetin and fisetin have been reported to increase the GSH level in various cancer cell lines [46,47,48]. A significant decrease in GSH levels has been detected in cervical cancer patients compared with healthy controls [44,49]. The treatment with fisetin of HeLa cells showed an increase in GSH level ≥ 3-fold, improving antioxidant stress (Figure 5A). It has been well established that increased ROS levels owing to inflammation trigger the production of pro-inflammatory cytokines, such as IL-1b, the IL-6 by activating NF-KB and JAK-STAT pathways [50]. In the current study, fisetin treatment significantly reduced the expression of various pro-inflammatory cytokines and chemokines at protein levels such as IL-1α, IL-1β, IL-7, IL-11 IL-16, IL-12p70, MIG, MCP-1, MCP-2, MIP-1β, MIP-1γ, MCF, I-309 and EOTAXIN compared with untreated control cells. In comparison, the expression of anti-inflammatory cytokines such as IL-10 and IL-13 were found to be upregulated (Table 1). Expression of TNF-α, s TNF RI and s TNF RII was also increased because TNF-α acts as a pro and anti-inflammatory cytokine and plays a significant role in inducing apoptosis (Figure 5B,C). Consistent with this finding, several studies documented that IL-10 impedes NF-*κ*B signalling and inhibits pro-inflammatory cytokine production [23,24,25,51,52,53]. Probably, fisetin-mediated upregulated expression of IL-10 and IL-13 (anti-inflammatory) cytokines inhibit NF-kB pathways by reducing the phosphorylation level of different proteins related to the pathway. Repressed NF-kB pathways directly or indirectly reduce the expression of various pro-inflammatory cytokines. Furthermore, fisetin treatment elevated total glutathione levels (GSH), which may further reduce inflammation by scavenging ROS and free radicals.

Fisetin treatment significantly modulated the phosphorylation of various molecular targets of NF-*κ*B and JAK-STAT pathway to reduce inflammation such as Src (P-Tyr419), NF-kB (P-Ser536), TAK1 (P-Ser412), TBK1 (P-Ser172) and HDAC2 (P-Ser394). Phosphorylation was also slightly reduced (Figure 6A,B). In line with this study, Luo et al. established that inhibiting the NF-*κ*B pathway led to tumour regression through TNF-*α*/TRAIL.

Many studies have shown similar results but with fewer phosphorylated proteins being examined. This report is among the first few to comprehensively examine the molecular consequences of fisetin-facilitated modulation of phosphorylated proteins that are associated with proliferation, apoptosis, oxidative stress, inflammation, invasion and metastasis (Table 2) [23,25,51,54].

The activated JAK–STAT pathway induces proliferation, cell survival and increases inflammation in cervical cancer. Activated JAK induces STAT phosphorylation and activation, which then can translocate to the nucleus and acts as a transcription factor. Therefore, their dephosphorylation will suppress the pathway [55,56]. The current study results demonstrate that fisetin treatment (50 µM) reduced or dephosphorylated STAT1 (P-Ser727), STAT2 (P-Tyr689), STAT3 (P-Tyr705), STAT5 (P-Tyr694) and TYK2 (P-Tyr1054) compared with the control; therefore, it represses the pathway. SOCS (suppressors of cytokine signalling) also regulates the activation of the JAK–STAT pathway [56]. This study showed an increased fold change of SOCS 1, which can additionally suppress the JAK–STAT pathway.

Activating PI3K/AKT and MAPK pathways through phosphorylation deters apoptosis; however, dephosphorylation probably leads to cell death [57,58,59]. PI3K–Akt and Ras–MAPK pathways congregate at BAD and mediate phosphorylation of BAD at two separate serine residues, i.e., serine-112 (Ser-112) and serine-136 (Ser-136). These two sites become phosphorylated by AKT and Ras–Raf (MAPK), respectively [12,60]. Phosphorylated BAD loses its ability to form a heterodimer with anti-apoptotic protein BCL-XL or BCL-2; therefore, it binds with 14-3-3 and is impounded in the cytoplasm, whereas the dephosphorylated BAD associates with pro-survival protein (BCL-XL or BCL-2) and accumulates in the mitochondrial membrane that subsequently upsurges apoptosis [12,60]. The AKT/PI3K pathway leads to activation of the mTOR and NF-*κ*B pathways. Notably, the activated Akt/PKB pathway inactivates pro-apoptotic genes such as Bad and caspase-9, FOXO-tumour suppressor gene (forkhead family of transcription factors) and FASL (pro-apoptotic factor), thereby leading to tumorigenesis. Therefore, targeting the AKT/PI3K pathway as a cancer therapy is currently employed to inhibit tumour progression [9,10,61]. In this study, fisetin treatment downregulated the AKT/PI3K/mTOR pathway by decreasing the expression of AKT2, MTOR, PIK3C2B and ELK 1 significantly, whereas PIK3CA and PIK3CB expression were slightly reduced at the transcript level. Fisetin treatment dephosphorylated various proteins involved in the AKT/PI3K/mTOR pathway, such as GSK3a, GSK3b, MTOR, PRAS 40, BAD (p-ser112), PTEN (p-ser380), AKT (p-ser473) and RPS6. In contrast, p27 (P-Thr198) expression was increased compared with the untreated control HeLa cells (Table 2) (Figure 6A,B). Phosphatase activity of PTEN increases by dephosphorylation at Ser380 and it directly represses the PI3K/AKT pathway by conversion of PIP3 back to PIP2 produced via PI3K [62]. Therefore, the result of this shows that fisetin dephosphorylates PTEN to suppresses the AKT/PI3K pathway.

The MAPK pathway is primarily responsible for cell proliferation, differentiation and cell survival. Ulixertinib BVD-523, a novel, reversible, ATP-competitive ERK1/2 inhibitor, has been reported to decrease cell growth and augment caspase activity in vitro. Similarly, in an in vivo xenograft, it revealed concentration-dependent growth inhibition and tumour regression. Fisetin brings about its anti-carcinogenic activity by substantial downregulation of multiple molecular targets of the MAPK pathway such as *MAPK1*, *MAK14*, *MAP2K6, MAP3K5* and ELK 1, with marginal reduction in MAP2K1 expression, whereas the expression of PTPRR (inhibitor of the MAPK pathway) was downregulated. Consistent with the transcript level, fisetin also reduced phosphorylated protein expression involved in the MAPK pathway. Certain proteins showed marginal alteration in fold change such as ERK1/ERK2, RSK1(P-Ser380) and CREB (P-Ser133), while Raf-1 (P-Ser301) showed a significant decrease. Phosphorylation levels of p38 (P-Thr180/Tyr182) and P53 (P-Ser15) were found to be significantly increased [8,35,63,64].

It was reported that p53 accumulation in protein levels induces phosphorylation of P53 at Ser15 residue, which increases the apoptosis in cancer cells [65]. Consistent with the previous reports, this study also established that fisetin upregulated both p53 and P53 (P-Ser15) expression. Polyphenol, such as luteolin and quercetin, have revealed a similar effect on AKT and MAPK pathways (Table 2) [13,30].

Plant-derived polyphenols, including fisetin, were acknowledged to have significant potential as an anti-carcinogenic candidate as they are capable of modulating multiple signalling pathways associated with cancer. However, the activity of this compound varies markedly in in vitro conditions when compared with in vivo responses. The low bioavailability of this compound could feasibly explain variation in the action of fisetin due to its hydrophobic nature. Several drug-loaded nanoparticles or micelle formulations have been attempted to enhance the bioavailability of fisetin, and most of them have exhibited higher anticancer efficacy than that of a fisetin solution alone [66,67,68]. Although most of the work reported on fisetin is based on in vitro and in vivo studies, preclinical study findings on the pharmacological properties of fisetin potentially demonstrate the necessity of conducting suitably designed clinical trials for humans [15]. These studies will provide definitive answers to the effectiveness of this appealing natural compound and offer new possibilities for the forthcoming clinical applications of fisetin in the near future.

## 4. Materials and Methods

### 4.1. Cell lines and Reagents 

HeLa cells (Human Cervical cancer) and AC-16 (Human cardiomyocyte), were gifted from Dr Mawieh, Sharjah university, Sharjah. HeLa and AC-16 cells were maintained in DMEM with 10% FBS (Sigma; St. Louis, MO, USA) with 1% penicillin and streptomycin procured from Sigma (Sigma; St. Louis, MO, USA) in a humidified environment with 5% CO_2_ at a constant temperature of 37 °C. 4′,6-diamidino-2-phenylindole (DAPI), dimethyl sulfoxide (DMSO), propidium iodide (PI), 3-(4, 5-dimethylthiazol-2-yl)-2, 5-diphenyl tetrazolium (MTT) and trypsin-EDTA were obtained from Sigma Aldrich (Merck KGaA).

### 4.2. Preparation of Drug Solutions 

Fisetin was procured from TOCRIS biosciences (Bristol, UK). In total, 69.87 mM fisetin solution was prepared in dimethyl sulphoxide, and aliquots were stored at −20 °C. Working dilutions between 1–70 µM in range were made in complete media for treatment.

### 4.3. Cell Viability Assay

Cytotoxicity of fisetin on HeLa and AC-16 cells was evaluated by MTT (thiazolyl blue tetrazolium bromide) assay. In total, ~1 × 10^4^ cells/well were seeded in 96 well plates and kept overnight at 37 °C. Next, the treatment started for 24 and 48 h with various concentrations of fisetin, i.e., 1, 10, 20, 30, 35, 40, 45, 50, 55, 60, 65 and 70 µM in complete media on HeLa cells while the AC-16 cells (cardio myocyte) were treated with several concentrations (1–400 µM) of fisetin for 24 h. All the treatments were conducted in triplicates. After 24 h and 48 h treatment, 10 µL/well of MTT (Sigma, USA) with a final concentration of 0.5 mg/mL in PBS was added to each well and incubated for 2–4 h at 37 °C. Subsequently, 100 µL of DMSO was added in each well to dissolve the formazan crystal. The absorbance reading was taken with Absorbance Microplate Reader (BioTek, USA) at 570 nm. The % of cell viability was determined with the help of the given equation.

Cell viability (%) = (OD of treated groups)/(OD of control groups) × 100(1)

The microscopic analysis of fisetin treated HeLa cells was evaluated by using an inverted microscope (Labomed, Los Angeles, CA, USA).

### 4.4. Nuclear Morphology by DAPI (4,6-diamidino-2-phenylindile) Staining

HeLa cells (~3 × 10^4^ cells/500 mL) were seeded in 24 well plates and kept overnight at 37 °C. After completing the treatment at two time points i.e., 24 and 48 h, respectively, PBS wash of the cells was performed followed by fixing the cells with 70% ice-cold ethanol for 20 min. Subsequently, the fixed cells were incubated with DAPI to stain the nucleus (2.5 µg/mL of PBS) for 15 min in the dark, followed by an examination of nuclear morphology with the help of a fluorescent microscope. All the images were captured at ×20.

### 4.5. DNA Fragmentation Assay 

Apoptosis induces nuclear DNA cleavage by nuclear endonuclease, which can be analysed by agarose gel electrophoresis. DNA fragmentation assay was performed using the Quick Apoptotic DNA Ladder Detection Kit by Ray biotech following manufacturer protocol to affirm fisetin-mediated nuclear cleavage. Briefly, ~1 × 10^6^ cells were plated in each flask and treated with fisetin (0, 20, 30 and 50 µM for 48 h). Cells were gently trypsinized and pelleted, followed by resuspension in lysis buffer. Then, the DNA was precipitated by isopropanol alcohol. The DNA with the suspension buffer was subjected to gel electrophoresis with 1.2% agarose comprising 0.5 mg/mL ethidium bromide in both gel and running buffer. The gel was visualized under a UV light transilluminator.

### 4.6. Cell Cycle Analysis 

Treated and untreated HeLa cells were used to analyse their DNA content using the Propidium Iodide Flow Cytometry Kit from Abcam (ab139418, Cambridge, UK). A total of ~2 × 10^6^ cells per concentration were treated with 0, 20, 30, and 50 µM of fisetin. It was then followed by the respective time point treatment, i.e., the cells were harvested through gentle trypsinization with 0.05% trypsin, fixed and kept overnight at −20 °C. Then, the cells were stained with PI as per the manufacturer’s protocol, followed by analysis for various cell cycle phases using flow cytometer (FACS Calibur; Becton-Dickinson, Franklin Lakes, NJ, USA). Data were analysed by FlowJo, Software (FlowJo LLC; version 10.1). The experiment has been repeated thrice.

### 4.7. Annexin V/Propidium Iodide Double Staining to Quantitate Apoptosis

The proportion of cells that have undergone apoptosis was determined by Annexin V-FITC Apoptosis detection kits (ab14085, Cambridge, UK). Treated HeLa cells (~2 × 10^5^ cells/well in a six-well plate) at a concentration of 20, 30 and 50 μM at a time point of 24 and 48 h were harvested and washed with PBS followed by staining with Annexin–Pi for 20 min in a dark room. The early and late apoptotic cells were determined by (FACS Calibur, Becton-Dickinson, Franklin Lakes, NJ, USA). 

### 4.8. TMRE Staining to Analyse Mitochondrial Membrane Potential 

TMRE (Tetramethylrhodamine, ethyl ester) dye was used to stain the live cell using TMRE-Mitochondrial Potential assay kit from Abcam (ab113852; Cambridge, UK). The kit was employed to analyse the impact of fisetin on alteration in mitochondrial membrane potential. The assay was performed as per the kit’s protocol. Briefly, ~5 × 10^3^ cells/well was treated with 20, 30 and 50 µM of fisetin for 48 h followed by the addition of TMRE in control and treated cells while FCCP was added to the negative control cells (FCCP prevents staining by TMRE). The plate was then incubated at 37 °C for 30 min, the fluorescence reading was taken by using microplate spectrophotometry (Ex/Em = 549/575 nm) and images were captured to examine the mitochondrial fluorescence intensity in treated and untreated controls by a fluorescence microscope (Progress Fluorescent Microscope Olympus, USA) at ×40.

### 4.9. Gene Expression by TaqMan Apoptosis Array

RNA was isolated from the treated (20, 30 and 50 µM for 48 h) and control cells as per the kit’s protocol (Gen Elute Mammalian Genomic Total RNA Kit; Sigma, USA). The qualitative check was performed by running the isolated RNA in 1% agarose gel and the same was quantitated using nanodrop (Nanodrop 2000c; Thermo Scientific™, Waltham, MA, USA). The RNA was used to synthesize cDNA with the help of a High-Capacity cDNA Reverse Transcription Kit (Applied Biosystems™, Waltham, MA USA) as per the manufacturer’s instruction. TaqMan^®^ Gene Expression Array and master mix (Apoptosis Array, cat. No. 4414072 and 4369514) was employed to evaluate the expression of various molecular targets involved in apoptosis and signalling pathways, including cell proliferation, cell survival, etc. To each well of the assay plate, 10 µL of cDNA (100 ng/well) from the treated cells, along with 10 µL of the master mix, was added. The plate was then subjected to run for qPCR (QuantiStudio3; Applied Biosystems) and analysis was performed by DataAssistTM software version 3.01 (ThermoFisher Scientific) with the 2^−^^ΔΔ^^Cq^ method. The expression level of GAPDH (a housekeeping gene) was used to normalize the data. The RQ values display the fold change for the expression of various genes in treated cells compared with the untreated control.

### 4.10. Measurement of Apoptosis-Related Proteins Expression by Proteome Profiler Array 

Modulated manifestation of proteins pertaining to apoptosis after treatment with 20 µM and 50 µM fisetin was ascertained by RayBio^®^ Human Apoptosis Arrays C1(Cat. No. AAH-APO) and was compared with the untreated control. Firstly, protein quantitation was conducted by Pierce BCA assay (Catalogue no: 23225; Thermo Fisher Scientific, USA). Each nitrocellulose membrane was incubated with 500 µg of the diluted protein sample from the lysate and kept overnight on a rocking platform at 4 °C. Then, the membranes were washed with wash buffers to discard any unbound proteins. Next, labelling was performed with biotinylated antibody followed by signal development through HRP-Streptavidin. It was then followed by thorough washing; the membranes were incubated with 500 µL of detection buffer for 2 min at room temperature. Within 5 min, the membranes were exposed to a chemiluminescent detection gel Doc device (Bio-Rad Laboratories; Richmond, CA, USA). The data analysis was conducted employing Image Lab software (version 6.0.1, Bio-Rad, Hercules, CA, USA).

### 4.11. Phosphorylation Array

Modulated expression of a variety of phosphorylated proteins followed by 50 µM fisetin treatment was evaluated by RayBio^®^ Human Phosphorylation Pathway Profiling Array C55 (AAH-PPP-1-2), and the expression of various molecular targets involved in different pathways was correlated with the untreated control. The experiment was carried out exactly as per the kit’s protocol, which was already explained under apoptosis array. Data analysis was completed through image lab software, version 6.0.1 (Bio-Rad, Richmond, CA, USA).

### 4.12. Caspases Multiplex Assay

To ascertain the type of apoptotic pathway employed by fisetin to induce cell death, caspase multiplex (Caspase 3, 8 and 9) assay was performed. The caspase multiplex assay kit (fluorometric) was obtained from Abcam (ab219915; Cambridge, UK), and the assay was completed according to the kit’s protocol. Briefly, ~8 × 10^3^ cells/per well were seeded in 96 well plate followed by treatment with various concentrations of fisetin (20, 30 and 50 μM) for 48 h. All treatments were performed in triplicates. After treatment, each well was incubated with 100 μL of caspase substrate (for all three caspases) for 1 h. Fluorescence reading was measured at wavelengths between 370–620 nm, followed by fold change calculation.

### 4.13. Detection of GSH Activity in HeLa Cells

To establish if fisetin treatment alleviates the oxidative stress in HeLa cells, a GSH assay was carried out. The GSH assay kit (colorimetric) was procured from Biovision (Catalog #K261) and was completed as per the manufacturer’s protocol. Briefly, the treated cells (20, 30 and 50 µM) and untreated control cells were collected in ice-cold PBS followed by lysis with glutathione buffer. Then 5% SSA (sulfosalicylic acid) was added and subjected to centrifuge at 8000× *g* for 10 min. Supernatant was used for glutathione assay. Then, the 96 well plate was incubated for 10 min at RT with 160 µL of reaction mix to generate NADPH followed by the addition of 20 μL of each sample solution and substrate. After the incubation, an absorbance reading was taken at 405 nm and GSH activity was calculated.

### 4.14. Inflammation Array 

To validate the anti-inflammatory role of fisetin, modulated expression of different anti-inflammatory proteins after fisetin treatment at 50 µM was compared with the untreated control. Human inflammation antibody array was carried out, which was procured from Abcam (ab134003; Cambridge, UK). To perform the array, firstly, protein quantitation of the cell lysate was performed by BCA assay. After blocking the membrane by blocking buffer for 30 min, each membrane was incubated with ~250 µg of lysate overnight on a rocking platform at 4 °C, followed by washing with wash buffer I and II to discard any unbound proteins. After the washes, the nitrocellulose membranes were labelled with biotinylated antibody. Finally, HRP-Streptavidin was added and left on the rocking surface for 2 h followed by washing and the subsequent addition of 500 µL detection buffer mixture C and D. Within 5 min, the membrane was exposed to chemiluminescent detector gel doc system (Bio-Rad Laboratories, Richmond, California, USA). Image Lab software, version 6.0.1, Bio-Rad was used to analyse the data.

### 4.15. Statistical Analysis

Data were analysed by using the GraphPad prism program (version 9.2.0) with either one-way or two-way analyses of variance, followed by Tukey’s HSD post-hoc test. All the data have been expressed as mean ± SD of at least 3 experiments. *p*-value < 0.05 was considered statistically significant.

## 5. Conclusions

The current study results reveal that fisetin modifies PI3K/AKT, MAPK, TGF-β/WNT, JAK-STAT and NF-kB pathways by modulating the expression of multiple molecular targets at both transcript and proteins levels. Modulation in expression of different proteins leads to cell growth inhibition, cell cycle arrest, DNA damage, attenuating oxidative stress and alleviating inflammation, inducing apoptosis. The current study establishes clear evidence about the multifaceted role of fisetin as an anticancer agent with its differential action towards tumour and normal cells; therefore, a safer profile [69].

## Figures and Tables

**Table 1:** Table showing expression of various genes and proteins being modulated by fisetin to carry out its anticancer effect.

Hall Mark	Molecular Target	Transcript Expression		Protein Expression	
		Upregulation	Downregulation	Upregulation	Downregulation
Apoptosis	Caspases	CASP9, CASP7, CASP3, CASP6, CASP4, CASP8AP2, CASP2, CASP1 and CASP8		Caspase-3 and Caspase-8	
	Pro-apoptotic gene	*APAF-1*, *BCL10*, *BCL2A1*, *BCL2L1*, *BCL2L13*, *BCL2L2*, *BAD*, *BAK1* and *BAX*, *BOK*, HTRA2, PYCARD, RIPK1, RIPK2, *BID*, PYCARD, RIPK2 and RIPK1.		Bad, Bax, Bid, Bim, P21, p53, p27, (ligand), cyt-c and HSP27.	
	Death receptors	FAS, *FASL,* CARD6, CARD9, CRADD, DEDD, *FADD, TNF, TNFRSFS10A and TNFRSFS10B*		Fas, Fas ligand, TNFRII, TNFα, TNF β, TNFRF, TNFSFS, TRAIL R1 to TRAIL R4.	
	Anti-apoptotic gene		*BCL2, MCL1, BIRC5, and NAIP*		Bcl-2, BCL-w, clap-2, HSP70, LIVIN, Survivin and XIAP.
Sustained cell proliferation	Cell cycle regulation		*CCNB1, CCNB2, CCNE2, CDKN2A and CDK4.*		
	Anti-proliferation and TSGs (Tumour suppressor genes)	*PTPRR, FOXO 1, FOXO 3*. ATM, ATF2 and TP53	*TERT*		
Inflammation and anti-oxidation			IL-2 and MYC,	IL-10 and IL-13	IL-1α, IL-1β, IL-4, IL-7, IL-11 IL-16, IL-12p70, MIG, MCP-1, MCP-2, MIP-1β, MIP-1γ, MCF, I-309 and EOTAXIN

**Table 2:** Table showing fisetin-mediated modulation of various signalling pathways by altered expression of different genes and phosphorylated proteins.

Hall Mark	Molecular Target	Transcript Expression		Protein Expression	
		Upregulation	Downregulation	Upregulation	Downregulation
Pathways:Anti-proliferation, anti-inflammation and apoptosis-inducing pathway	MAPK	MAPK1, MAK14, MAP2K1, MAP2K6, ELK 1		p38 (P-Thr180/Tyr182) and P53 (P-Ser15)	RSK1 (P-Ser380) and Raf-1 (P-Ser301)
	AKT/MTPR/PI3K	AKT2, MTOR, PIK3C2B, PIK3CB.		p27 (P-Thr198)	GSK3a (p-ser21), GSK3b (p-ser9), MTOR (p-ser2448), PRAS 40 (p-Ther246), BAD (p-ser112), PTEN (p-ser380), AKT (p-ser473), AMPKa (P-Thr172), RPS6 (P-Ser235/236), and 4E-BP1 (P-Thr36).
	JAK-STAT and NF-kB				Src (P-Tyr419), STAT1 (P-Ser727), STAT2 (P-Tyr689), STAT3 (P-Tyr705), STAT5 (P-Tyr694), TYK2 (P-Tyr1054), HDAC4 (P-Ser632), NF-kB (P-Ser536), TAK1 (P-Ser412) and TBK1 (P-Ser172).

## Data Availability

Data is contained within the article. The data presented in this study are available in manuscript itself.

## Abbreviations

TNF	Tumour necrotic factor
FASL	Fas ligand
TRAIL	Tumour necrosis factor-related apoptosis-inducing ligand
PARP	Poly [ADP ribose] polymerase 1
PI	propidium iodide
PI3KCD	phosphotidyl-inositol-4,5-bisphosphate 3-kinase catalytic subunit delta
PTPRR	protein tyrosine phosphatase receptor type R
qPCR	quantitative real time polymerase chain reaction
TERT	telomerase reverse transcriptase
IL	Interleukin
MAPK	Mitogen activated protein kinase

## References

[1] Amararathna M., Johnston M.R., Rupasinghe H.P. (2016). Plant polyphenols as chemopreventive agents for lung cancer. Int. J. Mol. Sci..

[2] Patterson S.L., Colbert Maresso K., Hawk E. (2013). Cancer chemoprevention: Successes and failures. Clin. Chem..

[3] Sundarraj K., Raghunath A., Perumal E. (2018). A review on the chemotherapeutic potential of fisetin: In vitro evidences. Biomed. Pharmacother..

[4] Lall R.K., Adhami V.M., Mukhtar H. (2016). Dietary flavonoid fisetin for cancer prevention and treatment. Mol. Nutr. Food Res..

[5] Mehta R.G., Murillo G., Naithani R., Peng X. (2010). Cancer chemoprevention by natural products: How far have we come?. Pharm. Res..

[6] Raj L., Ide T., Gurkar A.U., Foley M., Schenone M., Li X., Tolliday N.J., Golub T.R., Carr S.A., Shamji A.F. (2011). Selective killing of cancer cells by a small molecule targeting the stress response to ROS. Nature.

[7] Sudhakar A. (2009). History of cancer, ancient and modern treatment methods. J. Cancer Sci. Ther..

[8] Lee S., Rauch J., Kolch W. (2020). Targeting MAPK signaling in cancer: Mechanisms of drug resistance and sensitivity. Int. J. Mol. Sci..

[9] Song M., Bode A.M., Dong Z., Lee M.-H. (2019). AKT as a therapeutic target for cancer. Cancer Res..

[10] Porta C., Paglino C., Mosca A. (2014). Targeting PI3K/Akt/mTOR signaling in cancer. Front. Oncol..

[11] Panji M., Behmard V., Zare Z., Malekpour M., Nejadbiglari H., Yavari S., Dizaj T.N., Safaeian A., Maleki N., Abbasi M. (2021). Suppressing effects of Green tea extract and Epigallocatechin-3-gallate [EGCG] on TGF-β-induced Epithelial-to-mesenchymal transition via ROS/Smad signaling in human cervical cancer cells. Gene.

[12] Scheid M.P., Schubert K.M., Duronio V. (1999). Regulation of Bad phosphorylation and association with Bcl-xL by the MAPK/Erk kinase. J. Biol. Chem..

[13] Kedhari Sundaram M., Raina R., Afroze N., Bajbouj K., Hamad M., Haque S., Hussain A. (2019). Quercetin modulates signaling pathways and induces apoptosis in cervical cancer cells. Biosci Rep..

[14] Samarghandian S., Azimi Nezhad M., Mohammadi G. (2014). Role of caspases, Bax and Bcl-2 in chrysin-induced apoptosis in the A549 human lung adenocarcinoma epithelial cells. Anti-Cancer Agents Med. Chem..

[15] Sun X., Ma X., Li Q., Yang Y., Xu X., Sun J., Yu M., Cao K., Yang L., Yang G. (2018). Anti-cancer effects of fisetin on mammary carcinoma cells via regulation of the PI3K/Akt/mTOR pathway: In vitro and in vivo studies. Int. J. Mol. Med..

[16] Chien C.-S., Shen K.-H., Huang J.-S., Ko S.-C., Shih Y.-W. (2010). Antimetastatic potential of fisetin involves inactivation of the PI3K/Akt and JNK signaling pathways with downregulation of MMP-2/9 expressions in prostate cancer PC-3 cells. Mol. Cell Biochem..

[17] Pal H.C., Sharma S., Elmets C.A., Athar M., Afaq F. (2013). Fisetin inhibits growth, induces G 2/M arrest and apoptosis of human epidermoid carcinoma A 431 cells: Role of mitochondrial membrane potential disruption and consequent caspases activation. Exp. Dermatol..

[18] Kang K.A., Piao M.J., Hyun J.W. (2015). Fisetin induces apoptosis in human nonsmall lung cancer cells via a mitochondria-mediated pathway. Vitr. Cell Dev. Biol..

[19] Smith M.L., Murphy K., Doucette C.D., Greenshields A.L., Hoskin D.W. (2016). The dietary flavonoid fisetin causes cell cycle arrest, caspase-dependent apoptosis, and enhanced cytotoxicity of chemotherapeutic drugs in triple-negative breast cancer cells. J. Cell Biochem..

[20] Li J., Cheng Y., Qu W., Sun Y., Wang Z., Wang H., Tian B. (2011). Fisetin, a Dietary Flavonoid, Induces Cell Cycle Arrest and Apoptosis through Activation of p53 and Inhibition of NF-Kappa B Pathways in Bladder Cancer Cells. Basic Clin. Pharmacol. Toxicol..

[21] Yang P.-M., Tseng H.-H., Peng C.-W., Chen W.-S., Chiu S.-J. (2012). Dietary flavonoid fisetin targets caspase-3-deficient human breast cancer MCF-7 cells by induction of caspase-7-associated apoptosis and inhibition of autophagy. Int. J. Oncol..

[22] Szliszka E., Helewski K.J., Mizgala E., Krol W. (2011). The dietary flavonol fisetin enhances the apoptosis-inducing potential of TRAIL in prostate cancer cells. Int. J. Oncol..

[23] Landskron G., De la Fuente M., Thuwajit P., Thuwajit C., Hermoso M.A. (2014). Chronic inflammation and cytokines in the tumor microenvironment. J. Immunol. Res..

[24] Zhang J.-M., An J. (2007). Cytokines, inflammation and pain. Int. Anesthesiol. Clin..

[25] Yahfoufi N., Alsadi N., Jambi M., Matar C. (2018). The immunomodulatory and anti-inflammatory role of polyphenols. Nutrients.

[26] Ying T.H., Yang S.F., Tsai S.J., Hsieh S.C., Huang Y.C., Bau D.T., Hsieh Y.H. (2012). Fisetin induces apoptosis in human cervical cancer HeLa cells through ERK1/2-mediated activation of caspase-8-/caspase-3-dependent pathway. Arch. Toxicol..

[27] Fu C.Y., Chen M.C., Tseng Y.S., Chen M.C., Zhou Z., Yang J.J., Lin Y.M., Viswanadha V.P., Wang G., Huang C.Y. (2019). Fisetin activates Hippo pathway and JNK/ERK/AP-1 signaling to inhibit proliferation and induce apoptosis of human osteosarcoma cells via ZAK overexpression. Environ. Toxicol..

[28] Xiao X., Zou J., Fang Y., Meng Y., Xiao C., Fu J., Liu S., Bai P., Yao Y. (2018). Fisetin and polymeric micelles encapsulating fisetin exhibit potent cytotoxic effects towards ovarian cancer cells. BMC Complement. Altern. Med..

[29] Pak F., Oztopcu-Vatan P. (2019). Fisetin effects on cell proliferation and apoptosis in glioma cells. Z. Nat. C.

[30] Raina R., Afroze N., Sundaram M.K., Haque S., Bajbouj K., Hamad M., Hussain A. (2021). Chrysin inhibits propagation of HeLa cells by attenuating cell survival and inducing apoptotic pathways. Eur. Rev. Med. Pharmacol. Sci..

[31] Kashyap D., Garg V.K., Tuli H.S., Yerer M.B., Sak K., Sharma A.K., Kumar M., Aggarwal V., Sandhu S.S. (2019). Fisetin and quercetin: Promising flavonoids with chemopreventive potential. Biomolecules.

[32] You Y., Wang R., Shao N., Zhi F., Yang Y. (2019). Luteolin suppresses tumor proliferation through inducing apoptosis and autophagy via MaPK activation in glioma. Onco Targets Ther..

[33] Youns M., Hegazy W.A.H. (2017). The natural flavonoid fisetin inhibits cellular proliferation of hepatic, colorectal, and pancreatic cancer cells through modulation of multiple signaling pathways. PLoS ONE.

[34] Huang L., Jin K., Lan H. (2019). Luteolin inhibits cell cycle progression and induces apoptosis of breast cancer cells through downregulation of human telomerase reverse transcriptase. Oncol. Lett..

[35] Zhang B., Gui L.S., Zhao X.L., Zhu L.L., Li Q.W. (2015). FOXO1 is a tumor suppressor in cervical cancer. Genet. Mol. Res..

[36] Zhang J., Ng S., Wang J., Zhou J., Tan S.-H., Yang N., Lin Q., Xia D., Shen H.-M. (2015). Histone deacetylase inhibitors induce autophagy through FOXO1-dependent pathways. Autophagy.

[37] Le Chatelier E., Nielsen T., Qin J., Prifti E., Hildebrand F., Falony G., Almeida M., Arumugam M., Batto J.-M., Kennedy S. (2013). Richness of human gut microbiome correlates with metabolic markers. Nature.

[38] Routy B., Le Chatelier E., Derosa L., Duong C.P.M., Alou M.T., Daillère R., Fluckiger A., Messaoudene M., Rauber C., Roberti M.P. (2018). Gut microbiome influences efficacy of PD-1-based immunotherapy against epithelial tumors. Science.

[39] Ehren J.L., Maher P. (2013). Concurrent regulation of the transcription factors Nrf2 and ATF4 mediates the enhancement of glutathione levels by the flavonoid fisetin. Biochem. Pharmacol..

[40] Naidu M.S.K., Suryakar A.N., Swami S.C., Katkam R.V., Kumbar K.M. (2007). Oxidative stress and antioxidant status in cervical cancer patients. Indian J. Clin. Biochem..

[41] Tavsan Z., Kayali H.A. (2019). Flavonoids showed anticancer effects on the ovarian cancer cells: Involvement of reactive oxygen species, apoptosis, cell cycle and invasion. Biomed. Pharmacother..

[42] Zahra K., Patel S., Dey T., Pandey U., Mishra S.P. (2021). A study of oxidative stress in cervical cancer-an institutional study. Biochem. Biophys. Rep..

[43] Birben E., Sahiner U.M., Sackesen C., Erzurum S., Kalayci O. (2012). Oxidative stress and antioxidant defense. World Allergy Organ. J..

[44] Rodgers E.H., Grant M.H. (1998). The effect of the flavonoids, quercetin, myricetin and epicatechin on the growth and enzyme activities of MCF7 human breast cancer cells. Chem. Biol. Interact..

[45] Moskaug J.Ø., Carlsen H., Myhrstad M.C.W., Blomhoff R. (2005). Polyphenols and glutathione synthesis regulation. Am. J. Clin. Nutr..

[46] Giovannini C., Filesi C., D’Archivio M., Scazzocchio B., Santangelo C., Masella R. (2006). Polyphenols and endogenous antioxidant defences: Effects on glutathione and glutathione related enzymes. Ann. Dell’istituto Super. Sanita.

[47] Mukundan H., Bahadur A.K., Kumar A., Sardana S., Naik S.L.D., Ray A., Sharma B.K. (1999). Glutathione Level and Its Relation to Radiation Therapy in Patients with Cancer of Uterine Cervix.

[48] Althunibat O.Y., Al Hroob A.M., Abukhalil M.H., Germoush M.O., Bin-Jumah M., Mahmoud A.M. (2019). Fisetin ameliorates oxidative stress, inflammation and apoptosis in diabetic cardiomyopathy. Life Sci..

[49] Briukhovetska D., Dörr J., Endres S., Libby P., Dinarello C.A., Kobold S. (2021). Interleukins in cancer: From biology to therapy. Nat. Rev. Cancer.

[50] Chen G.Y., Shaw M.H., Redondo G., Núñez G. (2008). Innate immune receptor nod1 protects the intestine from inflammation-induced tumorigenesis. Cancer Res..

[51] Song Z., Lin Y., Ye X., Feng C., Lu Y., Yang G., Dong C. (2016). Expression of IL-1α and IL-6 is associated with progression and prognosis of human cervical cancer. Med. Sci. Monit. Int. Med. J. Exp. Clin. Res..

[52] Luo J.-L., Maeda S., Hsu L.-C., Yagita H., Karin M. (2004). Inhibition of NF-κB in cancer cells converts inflammation-induced tumor growth mediated by TNFα to TRAIL-mediated tumor regression. Cancer Cell.

[53] Brooks A.J., Putoczki T. (2020). JAK-STAT Signalling Pathway in Cancer. Cancers.

[54] Gutiérrez-Hoya A., Soto-Cruz I. (2020). Role of the JAK/STAT pathway in cervical cancer: Its relationship with HPV E6/E7 oncoproteins. Cells.

[55] Zhang X.-J., Jia S.-S. (2016). Fisetin inhibits laryngeal carcinoma through regulation of AKT/NF-κB/mTOR and ERK1/2 signaling pathways. Biomed. Pharmacother..

[56] Li J., Gong X., Jiang R., Lin D., Zhou T., Zhang A., Li H., Zhang X., Wan J., Kuang G. (2018). Fisetin inhibited growth and metastasis of triple-negative breast cancer by reversing epithelial-to-mesenchymal transition via PTEN/Akt/GSK3β signal pathway. Front. Pharmacol..

[57] Fang X., Yu S., Eder A., Mao M., Bast R.C., Boyd D., Mills G.B. (1999). Regulation of BAD phosphorylation at serine 112 by the Ras-mitogen-activated protein kinase pathway. Oncogene.

[58] Hayakawa J., Ohmichi M., Kurachi H., Kanda Y., Hisamoto K., Nishio Y., Adachi K., Tasaka K., Kanzaki T., Murata Y. (2000). Inhibition of BAD phosphorylation either at serine 112 via extracellular signal-regulated protein kinase cascade or at serine 136 via Akt cascade sensitizes human ovarian cancer cells to cisplatin. Cancer Res..

[59] Yang J., Nie J., Ma X., Wei Y., Peng Y., Wei X. (2019). Targeting PI3K in cancer: Mechanisms and advances in clinical trials. Mol. Cancer.

[60] Nakahata S., Ichikawa T., Maneesaay P., Saito Y., Nagai K., Tamura T., Manachai N., Yamakawa N., Hamasaki M., Kitabayashi I. (2014). Loss of NDRG2 expression activates PI3K-AKT signalling via PTEN phosphorylation in ATLL and other cancers. Nat. Commun..

[61] Germann U.A., Furey B.F., Markland W., Hoover R.R., Aronov A.M., Roix J.J., Hale M., Boucher D.M., Sorrell D.A., Martinez-Botella G. (2017). Targeting the MAPK signaling pathway in cancer: Promising preclinical activity with the novel selective ERK1/2 inhibitor BVD-523 [ulixertinib]. Mol. Cancer Ther..

[62] Hsieh M.-H., Tsai J.-P., Yang S.-F., Chiou H.-L., Lin C.-L., Hsieh Y.-H., Chang H.-R. (2019). Fisetin suppresses the proliferation and metastasis of renal cell carcinoma through upregulation of MEK/ERK-targeting CTSS and ADAM9. Cells.

[63] Choi E.J., Ahn W.S. (2008). Kaempferol induced the apoptosis via cell cycle arrest in human breast cancer MDA-MB-453 cells. Nutr. Res. Pract..

[64] Ghosh P., Roy A.S., Chaudhury S., Jana S.K., Chaudhury K., Dasgupta S. (2016). Preparation of albumin based nanoparticles for delivery of fisetin and evaluation of its cytotoxic activity. Int. J. Biol. Macromol..

[65] Feng C., Yuan X., Chu K., Zhang H., Ji W., Rui M. (2019). Preparation and optimization of poly [lactic acid] nanoparticles loaded with fisetin to improve anti-cancer therapy. Int. J. Biol. Macromol..

[66] Kadari A., Gudem S., Kulhari H., Bhandi M.M., Borkar R.M., Kolapalli V.R.M., Sistla R. (2017). Enhanced oral bioavailability and anticancer efficacy of fisetin by encapsulating as inclusion complex with HPβCD in polymeric nanoparticles. Drug Deliv..

[67] Mehta P., Pawar A., Mahadik K., Bothiraja C. (2018). Emerging novel drug delivery strategies for bioactive flavonol fisetin in biomedicine. Biomed. Pharmacother..

[68] Pawar A., Singh S., Rajalakshmi S., Shaikh K., Bothiraja C. (2018). Development of fisetin-loaded folate functionalized pluronic micelles for breast cancer targeting. Artif. Cells Nanomed. Biotechnol..

[69] Raina R., Pramodh S., Rais N., Haque S., Shafarin J., Bajbouj K., Hamad M., Hussain A. (2021). Luteolin inhibits proliferation, triggers apoptosis and modulates Akt/mTOR and MAP kinase pathways in HeLa cells. Oncol. Lett..

